# Evaluation of Black Tea Polyphenol Extract Against the Retrogradation of Starches from Various Plant Sources

**DOI:** 10.3390/molecules17078147

**Published:** 2012-07-06

**Authors:** Huaxi Xiao, Qinlu Lin, Gao-Qiang Liu, Fengxiang Yu

**Affiliations:** 1 National Engineering Laboratory for Rice and By-product Further Processing, Central South University of Forestry & Technology, Changsha 410004, China; Email: xiaoxijingjing@163.com (H.X.); 2 Faculty of Food Science and Engineering, Central South University of Forestry & Technology, Changsha 410004, China; Email: phdqlin@yahoo.cn (Q.L.); 3 College of Life Science and Technology, Central South University of Forestry & Technology, Changsha 410004, China; 4 Department of Food Science and Technology, Hunan Biological Electromechanical Polytechnic, Changsha 410127, China; Email: yufengxiang_1@163.com

**Keywords:** black tea polyphenol extract, rice starch, maize starch, potato starch, retrogradation

## Abstract

The effects of black tea polyphenol extract (BTPE) on the retrogradation of starches from different plant sources were studied using differential scanning calorimetry (DSC) and X-ray diffraction (XRD). DSC analysis shows that the gelatinization temperature of maize starch and starches from different rice varieties increased with increasing BTPE level. After storage at 4 °C, BTPE at a concentration of 15% markedly retarded the retrogradation of maize starch and starches from different rice varieties. Native maize starch and starches from different rice varieties showed typical A-type X-ray diffraction patterns, while native potato starch showed a typical B-type X-ray diffraction pattern. Adding BTPE significantly affected the crystalline region and intensities of X-ray diffraction peaks of maize and rice starch granules. It is concluded that adding BTPE markedly inhibits the retrogradation of maize starch and starches from different rice varieties, but has no significant influence on the gelatinization and retrogradation characteristics of potato starch.

## 1. Introduction

The starch granule is semicrystalline. When heated in the presence of water, the semicrystalline structure in granules transforms to an amorphous form, a process termed gelatinization. Gelatinized starch, however, tends to re-associate into an ordered crystalline structure during storage, which is called retrogradation [1]. As the retrogradation of starch affects the produce characteristics and shelf life of starchy foods, the inhibition of retrogradation of starch have been extensively investigated by food scientists. The degree of starch retrogradation and the properties of the starch crystallites formed are influenced not only by the storage time and temperature, but also by starch concentration [2,3], the botanical origin of the starch, molecular ratio of amylose to amylopectin and structures of amylose and amylopectin molecules [4,5]. Industrially, starch is subjected to various chemical or physical modifications to prevent starch retrogradation [6,7,8]. However, many chemical modifications used in food products are not safe due to the use of questionable chemicals. 

Tea is a water extract of the leaves of the plant *Camellia sinensis*. It has attracted significant attention recently, both in the scientific and in consumer communities, for its health benefits for a variety of disorders, ranging from cancer to weight loss. Tea leaves are processed differently to give green, black, or oolong tea. Green tea constitutes about 20% of the tea manufactured in the World, oolong tea is about 2% and black tea is nearly 78% [9]. It is well known that polyphenolic compounds, particularly the catechins, are the main compounds of tea. This group of polyphenols, present in oolong tea, black tea and green tea, is called the tea catechins. Easily oxidized during the fermentation process with the aid of enzymes, tea catechins are mainly retained in green tea, which is a nonfermented product [10]. However black teas are fully fermented, and enzymatic oxidation (by polyphenol oxidase) during black tea manufacture converts catechins into dimers and polymers such as theaflavins. 

Many studies have demonstrated that due to their strong free radical scavenging ability, tea polyphenols exhibit antibacterial and antioxidative activities, and show good prospects for their use as preservatives and antioxidants [11,12]. In recent years, a few studies on the relationship between starch and tea polyphenols have been reported. Zhu *et al.* [13] studied the effects of extracts of phytochemicals from green tea on the pasting, thermal, and gelling properties of wheat starch. Our previous studies investigated the effect of green tea polyphenols on the retrogradation of a high amylose rice starch [14], and the inhibitory effects of green tea polyphenols on the retrogradation of starches from different botanical sources [15]. However, data on the interaction and association between black tea polyphenols and retrogradation of maize, potato and rice starches are scarce. This paper aims at investigating the effects of black tea polyphenol extract (BTPE) on the retrogradation of starches from different plant sources (maize, rice and potato) and starches from different rice varieties.

## 2. Results and Discussion

### 2.1. Amylose and Moisture Contents of Starches from Various Plant Sources

The amylose contents in maize starch (MS), potato starch (PS) and rice starch (RS, the same as high amylose rice starch, HAR) were 27.6%, 20.3% and 28.3%, respectively, while the amylose contents in intermediate amylose rice starch (IAR) and low amylose rice starch (LAR) were 13.7% and 1.15%, respectively (Table 1). There were significant differences in amylose contents for starches from different rice varieties (HAR, IAR and LAR). The moisture contents of these starches ranged from 11.2 to 13.5% (Table 1).

**Table 1:** Amylose and moisture contents of starches from various plant sources.

Starches	Amylose (%)	Moisture (%)
MS	27.6 ± 0.13 a	11.6 ± 0.21 c
PS	20.3 ± 0.56 b	10.8 ± 0.24 d
RS (HAR)	28.3 ± 0.27 a	11.2 ± 0.17 c
IAR	13.7 ± 0.36 e	12.6 ± 0.26 b
LAR	1.15 ± 0.12 g	13.5 ± 0.18 a

Data are expressed as mean ± standard deviation. Values followed by the same letter in the same column are not significantly different (*p* < 0.05). MS, maize starch; PS, potato starch; RS (HAR), rice starch (high amylose rice starch); IAR and LAR (intermediate and low amylose rice starch).

### 2.2. Polyphenols in BTPE Sample

The polyphenol constituents in the BTPE sample are shown in Table 2. EGCG was the highest component of catechins in BTPE. Theaflavins was contained in high amounts. The total content of polyphenols of BTPE was 33.3%.

**Table 2:** Polyphenols in black tea polyphenol extract (BTPE) sample.

Polyphenols	% in Black Tea Polyphenol Extract
(−)-epigallocatechin gallate (EGCG)	13.8 ± 0.41
(−)-epicatechin gallate (ECG)	8.3 ± 0.32
(−)-epigallocatechin (EGC)	1.8 ± 0.11
(−)epicatechin (EC)	1.1 ± 0.04
(−)-gallocatechin gallate (GCG)	0.2 ± 0.02
(−)-catechin gallate (CG)	1.2 ± 0.03
Theaflavins	6.9 ± 0.42

Data are expressed as mean ± standard deviation.

### 2.3. Effect of BTPE on the Thermal Properties of Starcahes from Different Botanical Sources

The gelatinization thermal properties of starches from different botanical sources with and without the addition of BTPE as investigated by DSC are shown in Table 3. BTPE added to starches at various ratios significantly influences the thermal properties of MS and RS, however it has no obvious effect on the thermal properties of PS. The gelatinization temperatures (*T*_o_, *T*_p_ and *T*_c_) of RS and MS significantly increased with increasing BTPE level, while the enthalpy value of gelatinization (Δ*H*_gel_) decreased significantly as the BTPE level increased. The addition of BTPE showed different effects on the R values of starches from different botanical sources. The R values of RS increased with the content of BTPE increasing, in contrast, that of MS decreased. The R values of the starches adding 0%, 5%, 10%, and 15% BTPEs ranged from 9.73 to 11.08 °C for RS, and 8.05 to 6.32 °C for MS, respectively. The gelatinization temperatures, R values, and Δ*H*_gel_ of PS had no significant change before and after the addtion. This difference in the R value suggests that the degrees of heterogeneity of crystallites within granules of the studied starches are different [16]. 

**Table 3:** Gelatinization thermal properties of the mixtures of different starch samples and black tea polyphenol extract (BTPE) at various ratios.

Starch Samples	*T*_o_ (°C)	*T*_p_ (°C)	*T*_c_ (°C)	Δ*H*_gel_ (J/g)	R (°C)
MS + 0% BTPE	66.53 ± 0.47 f	71.03 ± 0.59 g	74.58± 0.79 f	10.62 ± 0.48 b	8.05 e
MS + 5% BTPE	68.05 ± 0.65 d	72.61 ± 0.65 e	76.03 ± 0.81 e	10.39 ± 0.42 b	7.98 e
MS + 10% BTPE	69.37 ± 0.54 b	73.85 ± 0.61 c	76.41± 0.85 d	9.23 ± 0.39 d	7.04 f
MS + 15% BTPE	71.28 ± 0.61 a	74.92 ± 0.69 a	77.60 ± 0.88 c	8.18 ± 0.33 e	6.32 g
PS + 0% BTPE	57.64 ± 0.32 g	62.40 ± 0.31 h	69.03 ± 0.67 g	12.14 ± 0.51 a	11.39 a
PS + 5% BTPE	57.68 ± 0.31 g	62.83 ± 0.32 h	69.00 ± 0.65 g	12.11 ± 0.51 a	11.32 a
PS + 10% BTPE	57.73 ± 0.30 g	62.84 ± 0.38 h	69.07 ± 0.63 g	12.17 ± 0.52 a	11.34 a
PS + 15% BTPE	57.69 ± 0.31 g	62.38 ± 0.35 h	69.09 ± 0.62 g	12.10 ± 0.53 a	11.40 a
RS (HAR) + 0% BTPE	66.39 ± 0.39 f	71.40 ± 0.55 f	76.12 ± 0.66 e	9.68 ± 0.32 c	9.73 d
RS (HAR) + 5% BTPE	67.35 ± 0.41 e	72.45 ± 0.57 e	77.65 ± 0.69 c	9.59 ± 0.27 c	10.30 c
RS(HAR) + 10% BTPE	68.41 ± 0.49 c	73.36 ± 0.62 d	78.88 ± 0.71 b	8.29 ± 0.24 e	10.47 c
RS(HAR) + 15% BTPE	69.27 ± 058 b	74.25 ± 0.58 b	80.35 ± 0.75 a	7.07 ± 0.19	11.08 b
IAR + 0% BTPE	64.33 ± 0.35 g	67.70 ± 0.37	71.64 ± 0.55	10.45 ± 0.43 b	7.31 g
IAR + 5% BTPE	65.17 ± 0.40 f	69.16 ± 0.48 g	73.08 ± 0.46 i	10.36 ± 0.38 b	7.91 f
IAR + 10% BTPE	66.90 ± 0.47 e	70.31 ± 0.52 f	74.91 ± 0.39 g	8.96 ± 0.25 d	8.01 f
IAR + 15% BTPE	67.34 ± 0.50 d	71.74 ± 0.59 e	75.83 ± 0.57 f	7.33 ± 0.23 e	8.49 e
LAR + 0% BTPE	65.03 ± 0.28 f	68.14 ± 0.47 h	72.51 ± 0.42 j	11.86 ± 0.49 a	7.48 g
LAR + 5% BTPE	66.14 ± 0.37 e	69.58 ± 0.38 g	73.90 ± 0.44 h	11.67 ± 0.41 a	7.76 f
LAR + 10% BTPE	67.29 ± 0.43 d	70.14 ± 0.53 f	75.26 ± 0.39 f	9.98 ± 0.33 c	7.97 f
LAR + 15% BTPE	68.61 ± 0.56 c	71.52 ± 0.56 e	77.40 ± 0.66 d	8.76 ± 0.27 d	8.79 e

The values are average ± standard deviation, n = 3. Values followed by the same letter in the same column are not significantly different (*p* < 0.05). R, the range of gelatinization (*T*_c_ − *T*_o_); MS, maize starch; PS, potato starch; RS (HAR), rice starch (high amylose rice starch); IAR and LAR (intermediate and low amylose rice starch); BTPE, black tea polyphenol extract.

After being stored at 4 °C for 5, 10, and 20 days respectively, the gelatinized samples of starches from different botanical sources, with or without the addition of BTPE, were reheated and rescanned in the DSC for examining their retrogradation thermal properties. It is well-known that the retrogradation starches reheated in a DSC showed lower transition temperature and enthalpy, because starch crystallinity had been weakened [17]. The results obtained in the second run heating DSC showed that the retrogradation enthalpies and retrogradation ratios of gelatinized RS and MS were decreased by the addition of BTPE, whereas BTPE had no obvious inhibitory effect on retrogradation of PS (Table 4). The retrogradation trend of all samples tested significantly increased with storage time increasing. The Δ*H*_ret_ values of RS with 10% and 15% BTPEs still could not be detected after 5 days of storage. Whereas the Δ*H*_ret_ values of MS with only 15% BTPE could not be detected after 5 days of storage, and the Δ*H*_ret_ value of RS and MS with 5% BTPE showed a little decrease in comparison with their native counterpart starches without the addition of BTPE (Table 4). The Δ*H*_ret_ values of RS and MS added BTPE all were detected after 10 or 20 days of storage. Whether 5 days storage and 10 days storage or 20 days storage, it was clearly shown that Δ*H*_ret_ and DR% of RS and MS began to decrease with increasing BTPE level, and BTPE at a concentration of 15% significantly retarded MS and RS retrogradation, however it was unable to retard PS retrogradation (Table 4). 

**Table 4:** The enthalpy and degree of retrogradation of mixtures of different starch samples and black tea polyphenol extract (BTPE) at various ratios after 4 °C storage.

Starch Samples	5 Days		10 Days		20 Days	
	Δ*H*_ret_ (J/g)	DR%	Δ*H*_ret_ (J/g)	DR%	Δ*H*_ret_ (J/g)	DR%
MS + 0% BTPE	3.14 ± 0.18 a	29.6 a	5.68 ± 0.45 a	53.5 a	8.01 ± 0.51 a	75.4 b
MS + 5% BTPE	3.07 ± 0.16 b	25.5 b	5.03 ± 0.41 b	48.4 c	7.52 ± 0.43 c	72.4 c
MS + 10% BTPE	1.87 ± 0.10 e	20.2 d	4.12 ± 0.36 c	44.6 d	6.49 ± 0.38 e	70.3 d
MS + 15% BTPE	ND	-	2.04 ± 0.28 e	24.9 h	4.54 ± 0.25 g	55.5 f
PS + 0% BTPE	2.68 ± 0.20 f	22.0 c	4.13 ± 0.34 c	34.0 g	7.48 ± 0.41 c	61.5 e
PS + 5% BTPE	2.66 ± 0.19 f	22.0 c	4.12 ± 0.35 c	34.0 g	7.42 ± 0.42 c	61.3 e
PS + 10% BTPE	2.45 ± 0.20 f	20.1 d	4.08 ± 0.33 c	33.5 g	7.46 ± 0.44 c	61.3 e
PS + 15% BTPE	2.36 ± 0.21 g	19.5 d	4.01 ± 0.32 c	33.1 g	7.43 ± 0.40 c	61.4 e
RS (HAR) + 0% BTPE	2.18 ± 0.19 c	22.5 c	4.98 ± 0.41 b	51.4 b	7.65 ± 0.44 b	79.0 a
RS (HAR) + 5% BTPE	2.01 ± 0.15 d	20.9 d	4.06 ± 0.38 c	42.3 e	6.91 ± 0.40 d	72.1 c
RS(HAR) + 10% BTPE	ND	-	3.15 ± 0.32 d	38.0 f	5.07 ± 0.39 f	61.2 e
RS(HAR) + 15% BTPE	ND	-	1.23 ± 0.11 f	17.4 i	2.36 ± 0.27 h	33.3 g
IAR + 0% BTPE	2.09 ± 0.16 b	20.0 d	3.43 ± 0.26 e	32.8 f	6.86 ± 0.48 d	65.6 d
IAR + 5% BTPE	1.97 ± 0.13 b	19.0 e	2.86 ± 0.24 f	27.6 g	5.74 ± 0.38 f	55.4 f
IAR + 10% BTPE	ND	-	2.03 ± 0.20 g	22.7 i	4.37 ± 0.29 g	48.8 g
IAR + 15% BTPE	ND	-	1.02 ± 0.11 h	13.9 k	2.08 ± 0.12 i	28.4 j
LAR + 0% BTPE	3.14 ± 0.24 a	26.5 a	6.43 ± 0.41 a	54.2 a	9.86 ± 0.61 a	83.1 a
LAR + 5% BTPE	3.02 ± 0.19 a	25.9 b	5.63 ± 0.39 b	48.2 c	8.51 ± 0.64 b	72.9 c
LAR + 10% BTPE	ND	-	4.27 ± 0.35 d	42.8 d	6.27 ± 0.45 d	62.8 e
LAR + 15% BTPE	ND	-	2.13 ± 0.21 g	24.3 h	3.44 ± 0.26 h	39.3 h

The values are average ± standard deviation, n = 3. Values followed by the same letter in the same column are not significantly different (*p* < 0.05). ND: not detectable; MS, maize starch; PS, potato starch; RS (HAR), rice starch (high amylose rice starch); IAR and LAR (intermediate and low amylose rice starch); BTPE, black tea polyphenol extract.

### 2.4. Evaluation of BTPE against the Retrogradation of Starches from Different Rice Varieties

The effect of BTPE on the thermal properties of HAR (the same as RS), IAR and LAR was further studied. The gelatinization transition temperatures and gelatinization enthalpy of native and added BTPE starches from different rice varieties are shown in Table 3. Among native rice starches, HAR had the highest gelatinization temperature and IAR had the lowest. However, the Δ*H*_gel_ value of HAR was the lowest only for 9.68 J/g and that of LAR was the highest for 11.86 J/g. HAR starch with longer average chain was reported to exhibit higher transition temperatures [18]. LAR had the highest heat of gelatinization due to being composed mostly of amylopectin, so its starch granule’s crystallinity is considered higher compared with those of HAR and IAR. Kreuger *et al*. [19] reported that more thermal energy was needed to initiate melting in the absence of amylose-rich amorphous regions. The R values of HAR adding 0%, 5%, 10%, and 15% BTPEs ranged from 9.73 to 12.28 °C, this was then followed by LAR and IAR with the R values ranging from 7.48 to 8.89 °C and 7.31 to 8.59 °C, respectively at the same adding ratios of BTPE. The result showed that BTPE resulted in an increase in R values of starches from different rice varieties was consistent with our previous study about green tea extract [20]. 

Table 3 also shows that the gelatinization temperatures (*T*o, *T*p and *T*c) increased significantly with the increasing content of BTPE. However, the enthalpy value of gelatinization (Δ*H*gel) decreased markedly as the BTPE level increased. The ΔHgel value ranged from 9.68 to 5.75 J/g for HAR, 10.45 to 6.11 J/g for IAR, and 11.86 to 7.04 J/g for LAR, respectively. 

After adding BTPE to MS and starches from different rice varieties, the increase in the gelatinization temperatures could be mainly contributed by the theaflavin content in BTPE. A study on the key polyphenols in teas indicated that theaflavins were present only in black tea samples [21]. As enzymatic fermentation proceeded, the content of theaflavins increased as the content of catechin decreased. Therefore, black teas had a relatively low amount of catechins. Several studies have examined the stability of tea catechins, and it was found that the stability of tea catechins was subject to heating temperature [22]. Under high temperature conditions, tea catechins were relatively unstable. The higher the temperature, the less stable the tea catechins. A turning point temperature of 82 °C was found to vary the stability of tea catechins [23]. In contrast to catechins, theaflavins had more stability towards heating due to their lower degree of polymerization compared to catechins, which may result in the increase in gelatinization temperatures of starches. The thermal properties of PS showed no obvious change after the addition of BTPE; it may be that PS had a higher phosphate monoester content compared with RS and MS, and phosphate monoester interacted with the hydroxyl (OH) groups of BTPE. On the other hand, PS had a bigger granule size in comparison with MS and RS [24]. 

When a starch gel stored at 4 °C is reheated in a DSC, an endothermic transition occurs that is not present in the DSC scan of the freshly gelatinized sample. The results obtained in the second run heating DSC showed that the retrogradation enthalpy and retrogradation ratio of gelatinized starches were significantly decreased by the addition of BTPE (Table 4). Current finding are in agreement with our previous report about green tea polyphenols [15]. However, constituent variation among the polyphenols of different teas significantly influences the inhibitory potency of retrogradation. Table 4 shows that the retrogradation trends of all samples tested significantly increased with increasing storage time. The Δ*H*_ret_ values of samples with 10% and 15% BTPEs still could not be detected after 5 days of storage, whereas the Δ*H*_ret_ values of samples with 5% BTPE were detected after 5 days of storage. After 20 days of storage, all samples showed retrogradation endotherms on the DSC. These results show that Δ*H*_ret_ and DR% began to decrease with the increasing concentration of BTPE, and BTPE at a concentration of 15% obviously retarded the retrogradation of HAR, IAR and LAR.

### 2.5. Effect of BTPE on X-ray Diffraction Patterns of Starches from Different Botanical Sources

**Figure 1:**
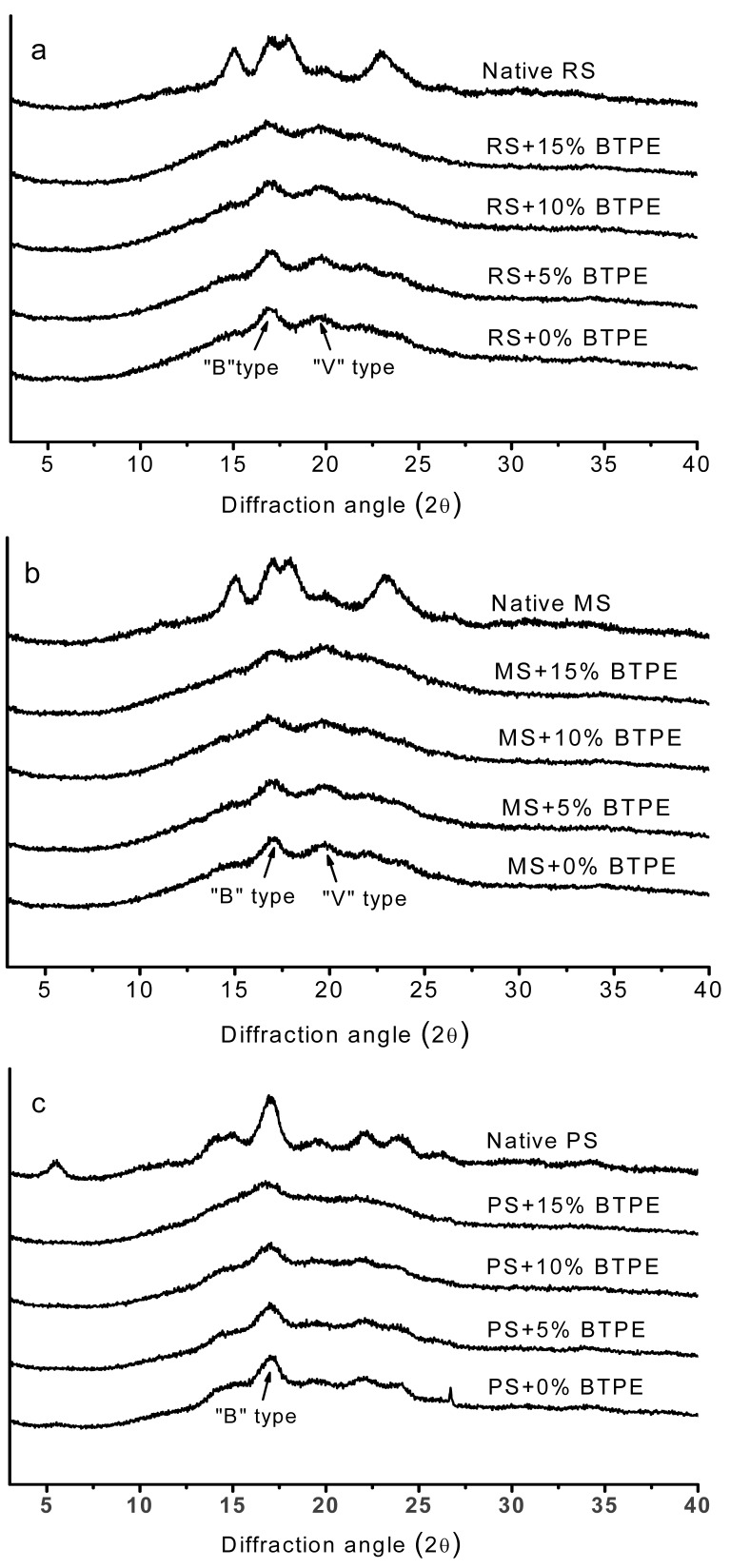
X-ray diffraction of native starch and gelatinized starch gels from different botanical sources containing different concentrations of black tea polyphenol extract (BTPE) after 10 days storage at 4 °C. (**a**) RS, rice starch; (**b**) MS, maize starch; (**c**) PS, potato starch.

X-ray diffraction patterns of native and retrograded starches from various botanical sources containing different concentrations of BTPE are shown in Figure 1. Normal native rice and maize starches (without retrogradation treatment) showed a typical A-type X-ray diffraction pattern with strong peaks at 2θ close to 15.4°, 17.3°, 18.5°, and 23.5° (Figure 1a,b), while normal native potato starch showed a typical B-type X-ray diffraction pattern (main peak at 2θ = 17.1°) (Figure 1c. 

After retrogradation treatment, rice and maize starches containing different concentrations of BTPE had a mixture of B-type and V-type (main peaks at 2θ = 19.68°) X-ray patterns, and potato starch containing different concentrations of BTPE showed similar diffraction patterns to native potato starch, *i.e*., the BTPE levels had no significant effect on the intensity of the diffraction peak in retrograded potato starch. This observation agrees with the previous results obtained using the DSC after 10 days of storage. The investigation using X-ray diffraction further proves the inhibiting effect of BTPE on the retrogradation of maize and rice starches.

### 2.6. Effect of BTPE on X-ray Diffraction Patterns of Starches from Different Rice Varieties

**Figure 2:**
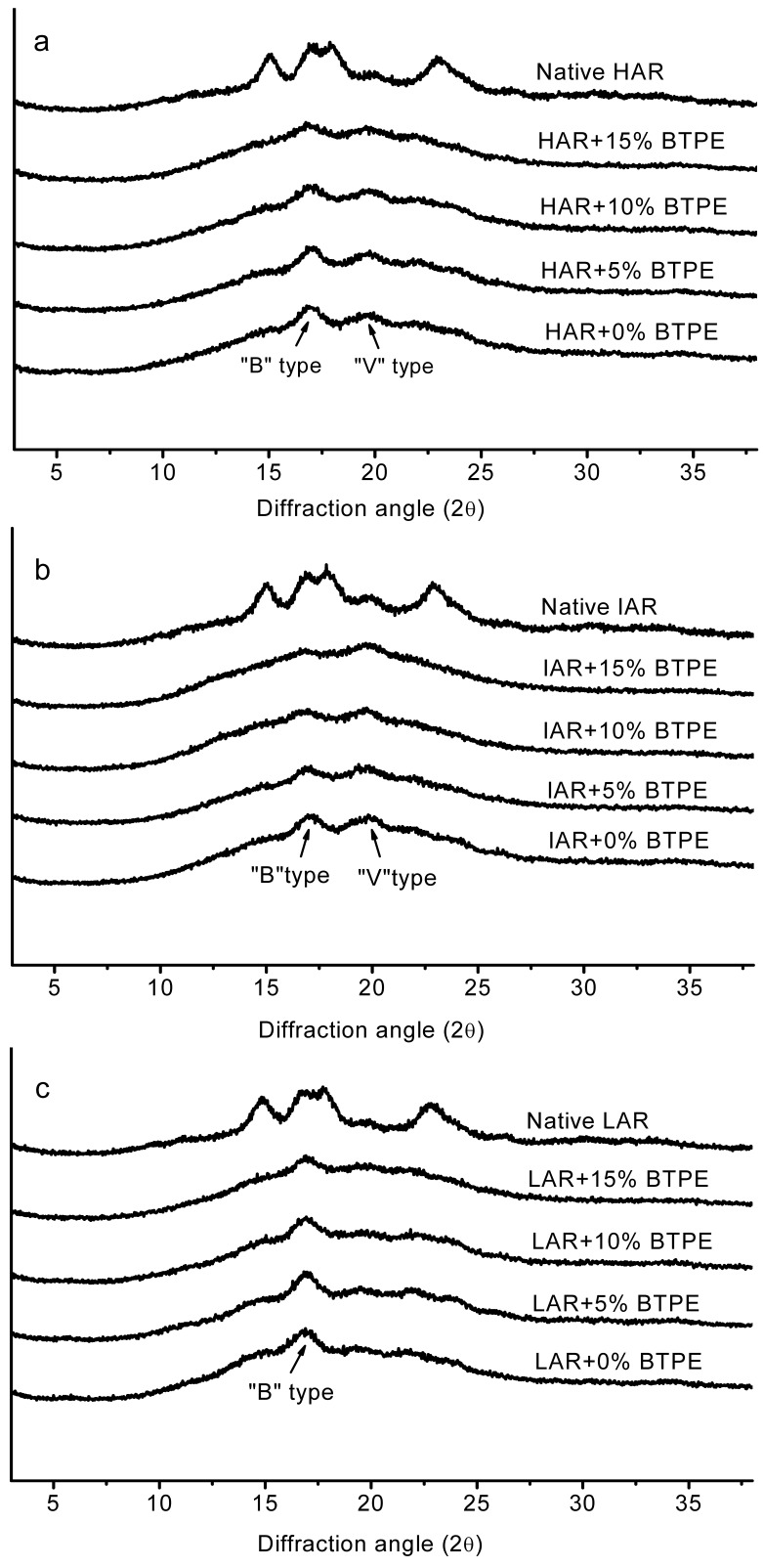
X-ray diffraction of native starch and gelatinized starch gels from different rice varieties containing different levels of black tea polyphenol extract (BTPE) after 10 days storage at 4 °C. (**a**) HAR, rice starch with high amylose content, the same as RS; (**b**) IAR, rice starch with intermediate amylose content; (**c**) LAR, rice starch with low amylose content.

Figure 2 shows the X-ray diffraction patterns and corresponding crystallinities of native starches and gelatinized starch gels from different rice varieties containing BTPE after 10 days of storage. It could be seen that the crystalline regions of HAR (the same as RS, Figure 2a, IAR (Figure 2b) and LAR (Figure 2c granules were obviously affected by the addition of BTPE. All the native starches from different rice varieties showed a typical A-type X-ray diffraction pattern, with strong peaks at 2θ close to 15.4, 17.3, 18.5, and 23.5. Retrograded LAR showed a B-type X-ray diffraction pattern, however retrograded HAR and IAR showed both B-type and V-type X-ray diffraction patterns.

The diffraction pattern of retrograded starch is clearly distinct from pattern of raw starch. Retrograded starch gives a B-type X-ray diffraction pattern due to the partial loss of granule crystallinity during heat treatment [25], which resulted in lower X-ray diffraction peak intensities in the samples. Amylose-lipid complexes are shown to possess V-type X-ray diffraction patterns with a peak at 2θ close to 20 [26]. All the diffractograms of HAR and IAR starches showed an obvious peak at approximately 20 (2θ), however none of the LAR starch diffractograms showed the characterized peak, which obviously is indicative of the amylose-lipid complex formation. Figure 2 shows that the addition of 5% and 10% BTPEs had no clearly effect on the intensities of X-ray diffraction peaks of all samples, whereas following the addition of 15% BTPE, the X-ray diffraction peaks intensities of all samples decreased significantly, implying that BTPE could inhibit the retrogradation behaviors of HAR, IAR and LAR, and further proving the conclusions from the DSC analysis results. 

## 3. Experimental

### 3.1. Materials

Maize starch (MS), potato starch (PS), high amylose rice starch (RS or HAR), intermediate amylose rice starch (IAR), and low amylose rice starch (LAR) were purchased from Pure Biological Technology Co. Ltd. (YunNan, China). BTPE was obtained as a fine brown yellow powder from Novanat Bioresources Co. Ltd. (Shanghai, China), Tea polyphenols, (–)-epigallocatechin gallate (EGCG), (–)-epigallo- catechin (EGC), (–)-epicatechin gallate (ECG), (–)-epicatechin (EC), (−)-gallocatechin gallate (GCG), (−)-catechin gallate (CG), and theaflavins were purchased from Sigma-Aldrich^®^ (St Louis, MO, USA). 

### 3.2. Determination of Amylose and Moisture Contents of Starches from Different Plant Sources

The amylose contents of rice, maize and potato starches were determined using the colorimetric method described by McGrance *et al.* [27]. The moisture contents of these starch samples were measured by heating at 105 °C for 5 h [15]. 

### 3.3. High Performance Liquid Chromatography (HPLC) Analysis of BTPE

The BTPE polyphenols were analyzed by high-pressure liquid chromatography (HPLC) with a DAD detector using a C_18_ reversed-phase column (250 × 4.6 mm × 5 μm, Waters, Milford, PA, USA). Mobile phases consisted of 0.1% methanoic acid in methanol (eluent A) and methanoic acid in water (eluent B). A gradient system was adopted from Wang and Zhou [28] with a slight modification: 0–10 min, 10% B; 10–30 min, linear gradient from 10% to 30% B; 30–36 min, linear gradient from 30% to 45% B; 36–46 min, linear gradient from 45% to 60% B; 46–50 min, 60% B; 50–55 min, linear gradient from 60% to 10% B. Post-run time was 5 min. The sample injection volume was 20 μL. The flow rate was 0.5 mL/min. Standard curves of peak area, as a function of the concentrations of EGCG, ECG, EGC, EC, GCG, CG and theaflavins were prepared for quantitative analysis. 

### 3.4. Differential Scanning Calorimetry (DSC)

Gelatinization and retrogradation thermal properties of the samples were measured using a 61 DSC-Pyris Diamond instrument (Perkin-Elmer Corp., Norwalk, CT, USA). Powdered starch and BTPE were mixed at the ratios of starch/BTPE for 2/0, 2/0.1, 2/0.2, and 2/0.3 (w/w) that equated with starch containing 0%, 5%, 10%, and 15% BTPEs (based on starch weight), respectively. The calorimeter was calibrated with an indium standard. Samples of the mixtures of starch and BTPE (about 2 mg) were accurately weighed into aluminum DSC pans, and deionized water was added by micropipette at a water-sample ratio of 2:1. The sample pans were sealed and equilibrated at room temperature for 24 h before analysis. For all DSC runs, a sealed empty aluminum pan was used as reference. The samples were heated. The scanning temperature range and the heating rate were 25–100 °C and 10 °C/min, respectively. The onset temperature *T*_o_, peak temperature *T*_p_ and conclusion temperature Tc were determined from the first run heating DSC curves, the range of gelatinization temperature (R = *T*_c_ − *T*_o_) was calculated. Enthalpy of gelatinization (Δ*H*_gel_) was evaluated based on the area of the main endothermic peak. Then the gelatinized samples were stored at 4 °C for 5, 10, and 20 days and rescanned under the same conditions to determine the temperature and enthalpy changes due to retrogradation. The retrogradation temperature (*T*_o_, *T*_p_ and *T*_c_) and enthalpy (Δ*H*_ret_) were determined from the second run heating. In addition, the degree of retrogradation (DR%) was calculated as the ratio of the retrogradation enthalpy to the gelatinization enthalpy in run heating [29]. Analyses were performed in triplicate. 

### 3.5. X-ray Diffraction

The samples as same as DSC analysis were rice, maize, and potato starches containing 0%, 5%, 10% and 15% BTPEs (based on starch weight). Double deionized water was added to the samples and gelatinized by steam heating for 20 min in a closed thermostatic water bath. These samples were cooled to room temperature then stored at 4 °C for 10 days. The freeze-dried samples were ground and powder passed through a 100 mesh sieve before testing. The recrystallization analysis of samples was carried out using a Rigaku D-Max-2500 X-ray diffractometer (Rigaku, Tokyo, Japan) equipped with a copper tube operating at 40 kV and 250 mA. Diffractograms were obtained, the scanning region of the diffraction angle (2θ) was from 4° to 50° at a rate of 4°/min, a step size of 0.02°, 1°/1° divergence slit/scattering slit, 0.30 mm receiving slit. MDI Jade 5.0 was used to analyze the diffractograms. 

### 3.6. Statistical Analysis

The data reported in the tables were average of triplicate observations. Data obtained were analyzed by analysis of variance (ANOVA) using SPSS for windows version 13.0. Confidence interval of sample means was reported at the 95% confidence probability. Comparisons of means were made using least significant difference (LSD) and shortest significant ranges (SSR) at 5% significance level (*p* < 0.05). 

## 4. Conclusions

The results of this work demonstrate that BTPE could inhibit the retrogradation of MS and starches with different amylose contents (HAR, IAR and LAR), but could not inhibit the retrogradation of PS. Further studies are required to elucidate the molecular mechanism that prevents retrogradation of these starches. 
